# Synthesis, Thermal Behavior, and Mechanical Properties of Fully Biobased Poly(Hexamethylene 2,5-Furandicarboxylate-*Co*-Sebacate) Copolyesters

**DOI:** 10.3390/polym15010085

**Published:** 2022-12-25

**Authors:** Shiwei Feng, Zhiguo Jiang, Zhaobin Qiu

**Affiliations:** State Key Laboratory of Chemical Resource Engineering, Beijing University of Chemical Technology, Beijing 100029, China

**Keywords:** biobased, crystallization, mechanical property, structure and property relationship

## Abstract

In this research, three fully biobased poly(hexamethylene 2,5-furandicarboxylate-*co*-sebacate) (PHFSe) copolyesters with low contents of hexamethylene sebacate (HSe) unit (10 mol%, 20 mol%, and 30 mol%) were successfully synthesized through a two-step transesterification/esterification and polycondensation method. The chemical structure and actual composition of PHFSe copolyesters were confirmed by hydrogen nuclear magnetic resonance. The thermal behavior and mechanical property of PHFSe copolyesters were investigated and compared with those of the poly(hexamethylene 2,5-furandicarboxylate) (PHF) homopolymer. Both PHFSe copolyesters and PHF showed the high thermal stability. The basic thermal parameters, including glass transition temperature, melting temperature, and equilibrium melting temperature, gradually decreased with increasing the HSe unit content. PHFSe copolyesters crystallized more slowly than PHF under both the nonisothermal and isothermal melt crystallization conditions; however, they crystallized through the same crystallization mechanism and crystal structure. In addition, the mechanical property, especially the elongation at break of PHFSe copolyesters, was obviously improved when the HSe unit content was greater than 10 mol%. In brief, the thermal and mechanical properties of PHF may be easily tuned by changing the HSe unit content to meet various practical end-use requirements.

## 1. Introduction

So far, the production and application of traditional petroleum-based plastics have caused severe environmental issues [1]. For instance, according to the 2021 IPCC Sixth Assessment Report (AR6), human activities, such as the massive extraction and abuse of oil, have led to a global temperature rising of about 1.09 °C since the industrial revolution. As a result, human-induced climate change has caused widespread adverse impacts far more than natural climate variability has [2]. These problems clearly indicate that the replacement of petro-based polyesters is extremely necessary [3,4,5,6]. It should be emphasized that poly(ethylene terephthalate) (PET) is one of the most used petro-based plastics at present.

The biomass-derived monomer 2,5-furandicarboxylic acid (FDCA) has obtained remarkable interests from both industries and academies in recent years [7,8]. Moreover, due to its similar chemical structure to that of terephthalic acid (TPA), poly(alkylene 2,5-furandicarboxylate)s (PAFs) are also considered as perfect biobased alternatives to PET and its homologues [9,10,11,12]. Poly(ethylene 2,5-furandicarboxylate) (PEF) is the one of the most promising polyesters among PAFs [13,14,15,16,17]. PEF has a high glass transition temperature (*T*_g_) of about 77 °C and a high melting temperature (*T*_m_) of around 214 °C, indicating that PEF shows excellent thermal properties [13]. Furthermore, PEF displays outstanding gas barrier properties, which is 11 times in O_2_ and 19 times in CO_2_ compared to those of PET [14,15]. However, both the poor toughness and the slow crystallization rate of PEF have seriously restricted its practical applications [13,16,17,18]. Poly(propylene 2,5-furandicarboxylate) (PPF) [19,20,21] and poly(butylene 2,5-furandicarboxylate) (PBF) [22,23,24] were the other two typical PAFs, both of which have been reported thoroughly. PPF shows a slow crystallization rate [19], while PBF shows a relatively fast crystallization rate [23].

Poly(hexamethylene 2,5-furandicarboxylate) (PHF) is one of the homologues of PEF [16,25,26,27]. Compared to that of PEF (4.2%), PHF shows a high elongation at break (*ε*_b_) of 210%, while it still possesses excellent thermal properties and strong crystallizability [16]. Papageorgiou et al. reported that PHF showed a *T*_g_ of 7 °C, a *T*_m_ of 145 °C, and an equilibrium melting temperature (*T*_m_°) of 157 °C [25]. PHF exhibited multiple melting behavior due to the melting, recrystallization, and remelting after isothermal crystallization. The gas barrier properties of PHF were slightly reduced compared to those of PEF, but were still better than those of PET [27].

Sebacic acid (SeA), a long-chain fatty acid extracted from castor oil, is a widely available and inexpensive biobased monomer. In a previous study, we synthesized and investigated the isothermal and nonisothermal crystallization kinetics of poly(ethylene succinate-*co*-sebacate) (PESSe) copolyesters [28]. Increasing the ethylene sebacate (ESe) content reduced both the crystallization rate parameter and the crystallization rate coefficient of PESSe copolyesters, suggesting that PESSe copolyesters with higher ESe content crystallized more slowly than those with lower ESe content. [28]. SeA is also often used as a third monomer to manipulate the mechanical property of polyesters [18,29]. In previous research, we prepared poly(butylene succinate)-*b*-poly (butylene sebacate) (PBS-*b*-PBSe) multiblock copolyesters. When the weight content of PBSe was about 40%, the *ε*_b_ increased significantly from 51.7% for PBS to 673.6% for PBS-*b*-PBSe [29]. Zhou et al. also prepared a series of poly(glycol furandicarboxylate-*co*-glycol sebacate) (PESF) copolyesters through introducing sebacic acid as a third monomer into PEF, which remarkably reduced the crystallinity and improved the *ε*_b_ of PEF [18].

Copolymerization is an easy way to regulate the thermal and mechanical properties of polymers [22,30,31,32,33,34,35]. A small amount of research on the copolymerization modification of PHF has been reported in the literature [8,36,37,38]. For instance, Kasmi et al. synthesized highly heat-resistant poly(hexamethylene-*co*-isosorbide-2,5-furandicarboxylates) copolyesters (PHIsF) [8]. The *T*_g_ values gradually increased from 10 to 135 °C with the addition of the third monomer, which enhanced the thermal properties of PHF. In a previous study, we synthesized poly(hexamethylene 2,5-furandicarboxylate-*co*-adipate) copolyesters (PHFA) [37]. Upon increasing the hexamethylene adipate (HA) unit content, the *T*_m_, *T*_m_°_,_ and crystallinity of PHFA gradually decreased; however, on the contrary, the *ε*_b_ remarkably increased to 498.3% for PHFA30 (HA mol% = 30 mol%) from 197.3% for PHF [38].

To the best of our knowledge, the synthesis and properties of fully biobased poly(hexamethylene 2,5-furandicarboxylate-*co*-sebacate) (PHFSe) copolyesters have not been reported in detail in the literature so far. In this research, we first synthesized PHFSe copolyesters using 1,6-hexanediol (HDO), SeA, and dimethyl 2,5-furandicarboxylate (DMFD) as the monomers; furthermore, we extensively investigated the thermal and mechanical properties of PHFSe copolyesters and compared them with those of PHF to clarify the influence of low contents of hexamethylene sebacate (HSe) unit. It is expected that the results reported herein should be interesting and important for a better understanding of the structure and property relationship of biobased polyesters from a sustainable viewpoint.

## 2. Experimental

### 2.1. Materials

The materials’ information, including the monomers (HDO, SeA, and DMFD) and the catalyst tetrabutyl titanate (TBT), is shown in the Supporting Information for brevity.

### 2.2. Synthesis of PHFSe Copolyesters

**Scheme 1:**
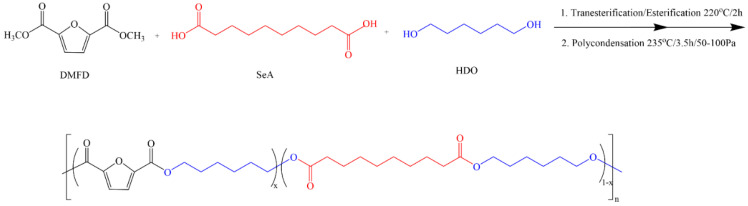
Synthesis route of PHFSe copolyesters.

PHFSe copolyesters were synthesized from DMFD, SeA, and HDO by a two-step melt polycondensation method [25,37]. The detailed synthesis procedure is also described in the Supporting Information for simplicity. Scheme 1 briefly describes the synthesis route. The copolyesters were abbreviated as PHFSe10, PHFSe20, and PHFSe30, with the number indicating the molar composition of the HSe unit. For comparison, PHF was also synthesized through a similar method.

### 2.3. Characterizations

The chemical structure, composition, thermal stability, basic thermal behavior, melt crystallization behavior, isothermal crystallization kinetics, crystal structure, and tensile mechanical property of PHFSe copolyesters were extensively studied with various techniques and compared with those of PHF. The detailed instruments and experimental conditions were similar to those described elsewhere in a previous study [37]. For brevity, they are also described in the Supporting Information.

## 3. Results and Discussion

### 3.1. Composition and [η] Values of PHF and PHFSe Copolyesters

**Figure 1:**
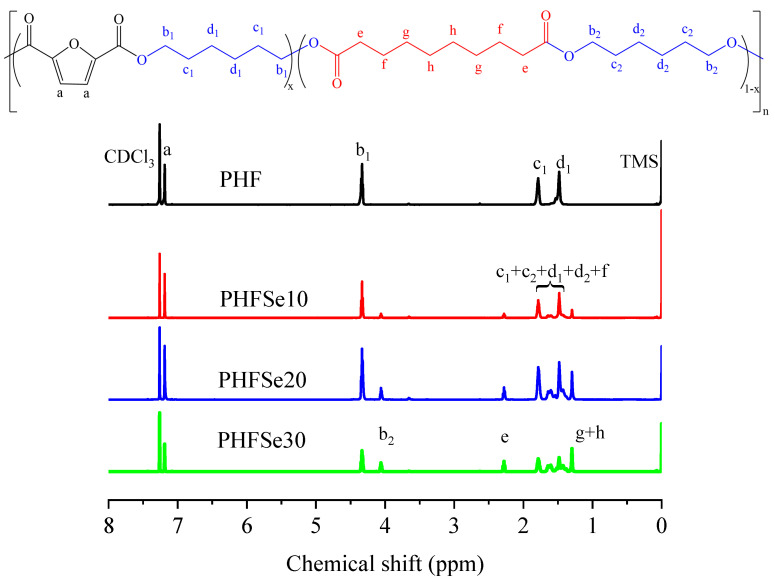
Chemical structure and ^1^H NMR spectra of PHF and PHFSe copolyesters.

The chemical structure and composition of PHF and PHFSe copolyesters were first determined with ^1^H NMR. Figure 1 depicts the related ^1^H NMR spectra. As shown in Figure 1, for the PHF homopolyester, the signal at 7.19 ppm was from the proton *a* of the furan ring, while the signals at 4.33 ppm, 1.78 ppm, and 1.48 ppm were from the protons *b*_1_, *c*_1_, and *d*_1_, respectively, of the hexamethylene 2,5-furandicarboxylate unit. In addition, for PHFSe copolyesters, the signals at 4.06 ppm (from the proton *b*_2_) and 1.60 ppm (from the protons *c*_2_ and *d*_2_) were from HDO in the HSe unit, while the signals at 2.28 ppm (from the proton *e*), 1.38 ppm (from the proton *f*), and 1.30 ppm (from the protons *g* and *h*) were from SeA in the HSe unit. The composition of PHFSe copolyesters was acquired by the integral ratio of *a* from DMFD and *e* from SeA. The compositions of the HSe unit in the as-synthesized copolyesters were calculated to be 10, 19, and 29 mol%, respectively. Since the molar ratios of HF/HSe were almost the same as those of DMFD/SeA, PHFSe copolyesters were successfully prepared. For convenience, the three copolymers were abbreviated as PHFSe10, PHFSe20, and PHFSe30, respectively, in terms of the feed molar ratio. The [*η*] of PHF and PHFSe copolyesters was calculated using the Solomon–Ciuta equation [39], and the [*η*] values of PHF, PHFSe10, PHFSe20, and PHFSe30 were 0.63, 0.68, 0.62, and 0.63 dL/g, respectively. The similar [*η*] values indicate that PHF and PHFSe copolyesters showed relatively high and similar molecular weights. The above data are recorded in Table 1.

### 3.2. Thermal Properties and Crystal Structure Studies

**Figure 2:**
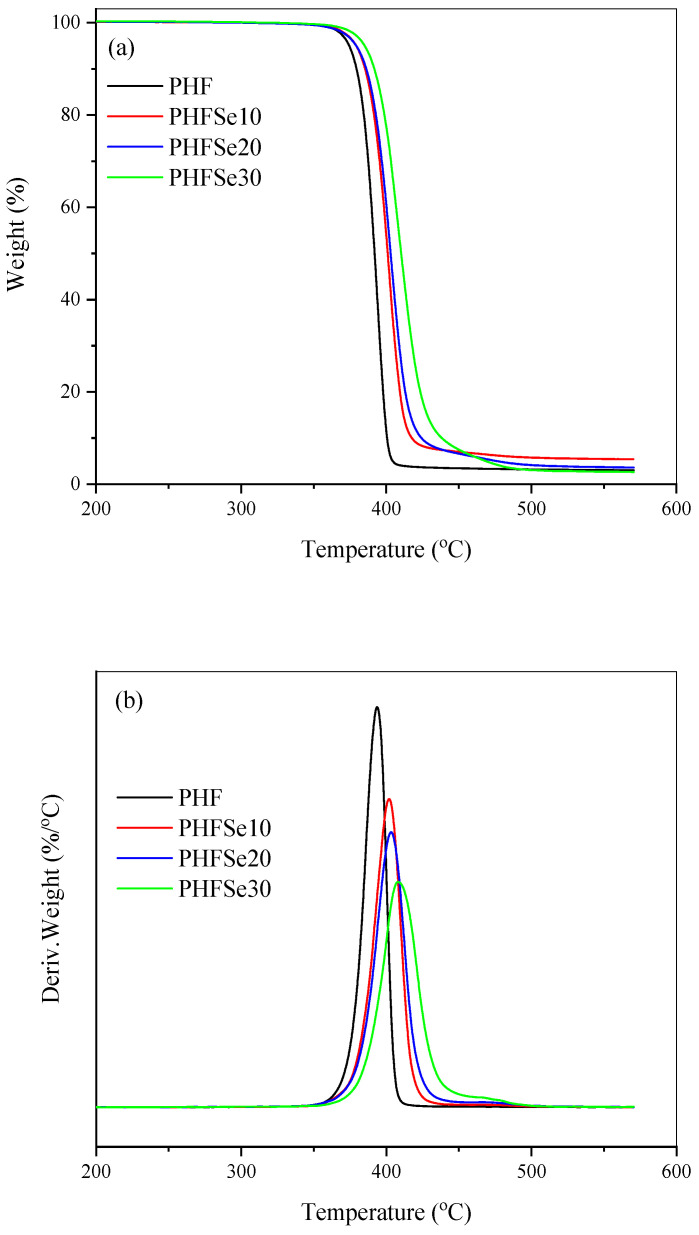
Thermal stability study of PHF and PHFSe copolyesters: (**a**) TGA and (**b**) DTG curves.

The thermal stability of polymeric materials is an important factor from polymer processing and practical application viewpoints. Figure 2 shows both the TGA curves and the derivative thermogravimetry (DTG) curves of PHF and PHFSe copolyesters at a heating rate of 20 °C/min under nitrogen atmosphere. The thermal decomposition temperature at weight loss of 5% (*T*_d_) values of all samples were close to or even above 375 °C, indicating that both PHF and PHFSe showed the good thermal stability. From Figure 2, all polyesters displayed the similar one-step thermal degradation process. As the HSe units content increased, the *T*_d_ values of copolyesters slightly increased; moreover, the temperature at maximum degradation rate (*T*_max_) shifted slightly to a higher temperature range. The related data are summarized in Table 2 for comparison.

**Figure 3:**
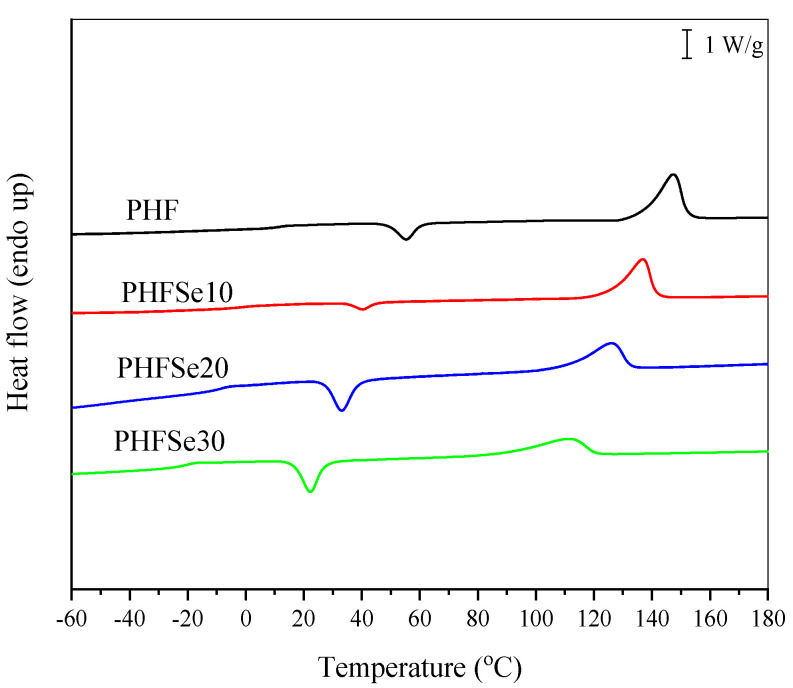
DSC heating curves of PHF and PHFSe copolyesters at 20 °C/min after quenching from molten state.

In this section, the basic thermal behavior of PHF and PHFSe copolyesters was studied with DSC. Figure 3 shows the DSC traces of PHF and PHFSe copolyesters at a heating rate of 20 °C/min after a melt-quenching process. The *T*_g_ of PHF was 12.3 °C, while the *T*_g_ of PHFSe copolyesters obviously shifted downward to a low temperature range with increasing the HSe unit content due to the increased the chain mobility. Both PHF and PHFSe polyesters showed the cold crystallization and subsequent melting behavior during the second heating process. The cold crystallization temperature (*T*_ch_) of PHF was 55.4 °C, while it gradually changed to 40.4 °C, 33.1 °C, and 22.3 °C for PHFSe10, PHFSe20, and PHFSe30, respectively. The cold crystallization enthalpy (Δ*H*_ch_) values were also measured. In addition, the *T*_m_ of PHFSe30 significantly decreased to 111.2 °C; furthermore, the melting enthalpy (Δ*H*_m_) decreased from 48.1 J/g for PHF to 35.3 J/g for PHFSe30 due to the copolymerization. As a result, the thermal properties of PHF may be obviously influenced and easily adjusted through copolymerization with SeA as the third comonomer in this research.

**Figure 4:**
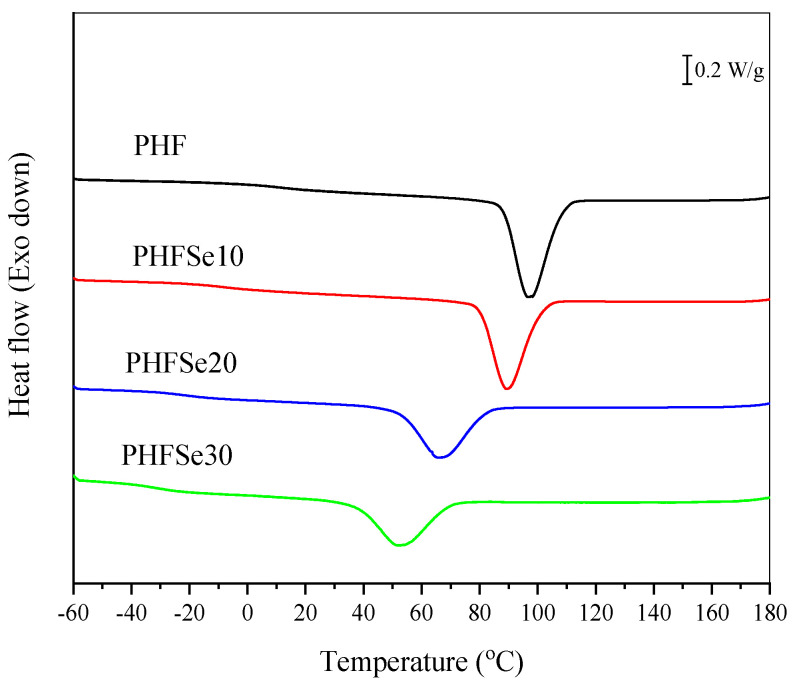
DSC cooling curves at 10 °C/min of PHF and PHFSe copolyesters.

DSC was also used to study the melt crystallization behavior of PHF and PHFSe copolyesters at a cooling rate of 10 °C/min. The relevant crystallization exotherms are illustrated in Figure 4. In the case of PHF, the melt crystallization temperature (*T*_cc_) and melt crystallization enthalpy (Δ*H*_cc_) were 97.2 °C and 52.6 J/g, respectively. In the case of PHFSe copolyesters, as the HSe units content increased, the *T*_cc_ apparently shifted downward to a lower temperature range; moreover, the Δ*H*_cc_ gradually decreased. For instance, the *T*_cc_ and Δ*H*_cc_ of PHFSe30 remarkably decreased to 51.8 °C and 36.5 J/g, respectively. The above results revealed that copolymerization with SeA greatly suppressed the crystallizability of PHF.

**Figure 5:**
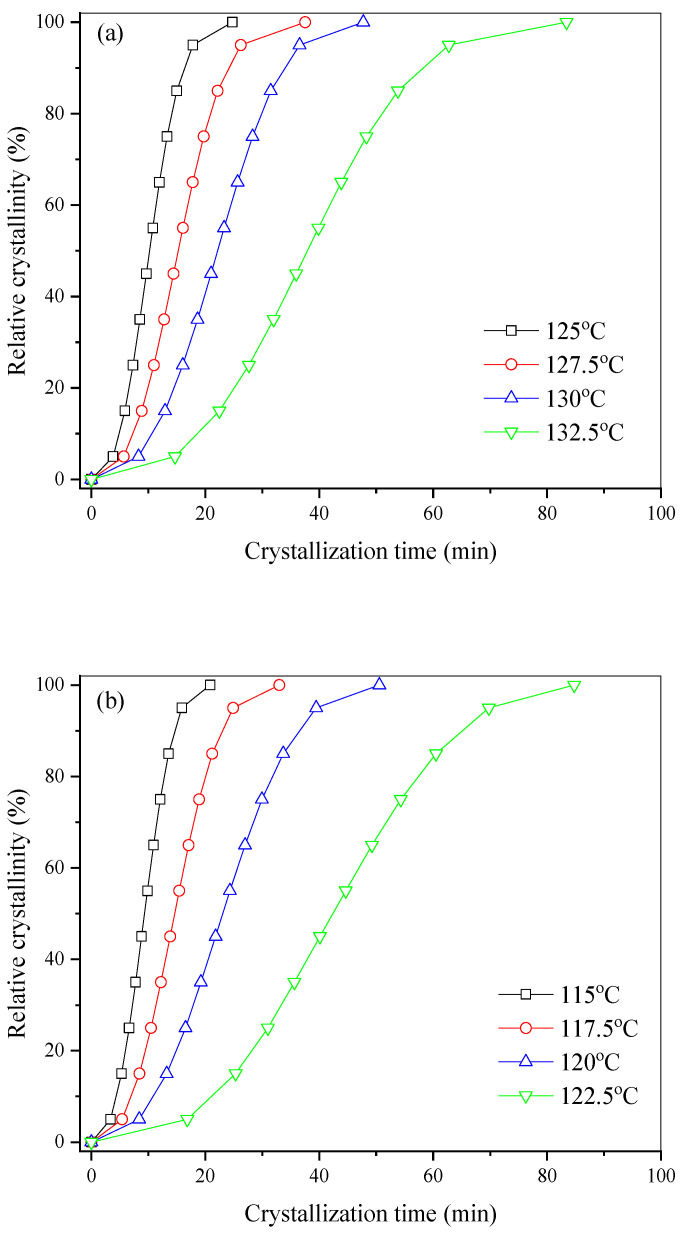

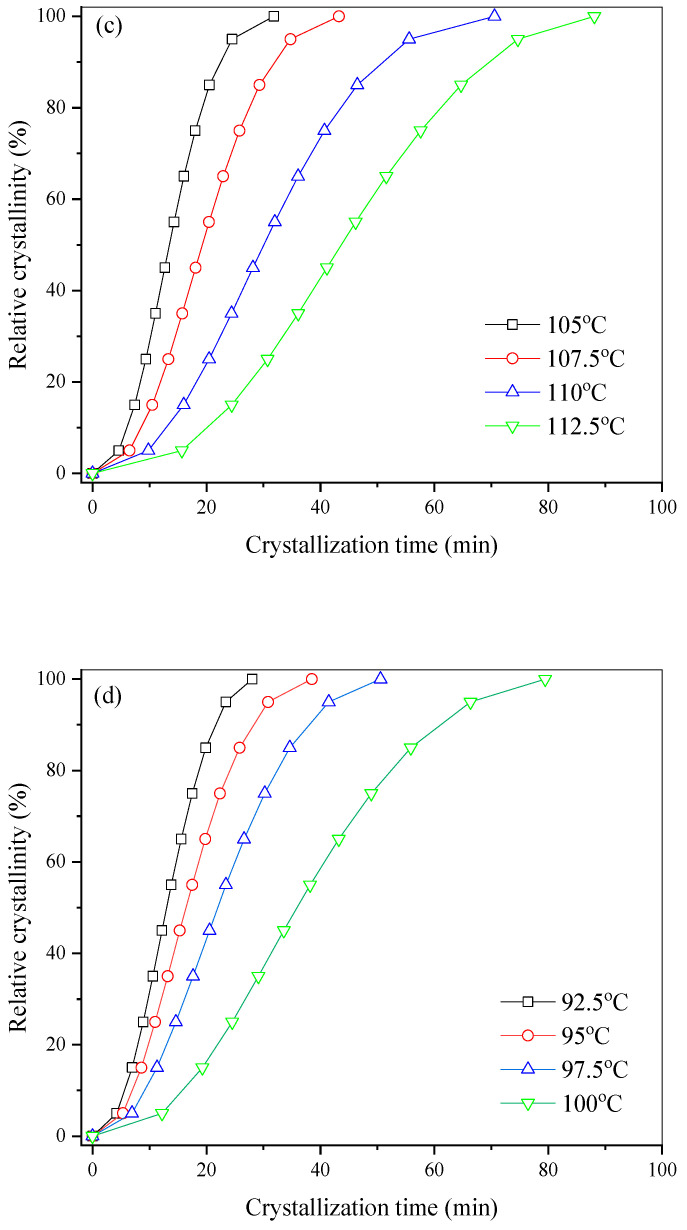
Plots of relative crystallinity versus crystallization time for (**a**) PHF, (**b**) PHFSe10, (**c**) PHFSe20, and (**d**) PHFSe30.

The isothermal melt crystallization kinetics of PHF and PHFSe copolyesters were further investigated with DSC. The plots of relative crystallinity versus time are displayed in Figure 5 for PHF and PHFSe copolyesters. As the degree of supercooling decreased, the crystallization time of all samples became obviously longer with an increase in crystallization temperature (*T*_c_).

**Figure 6:**
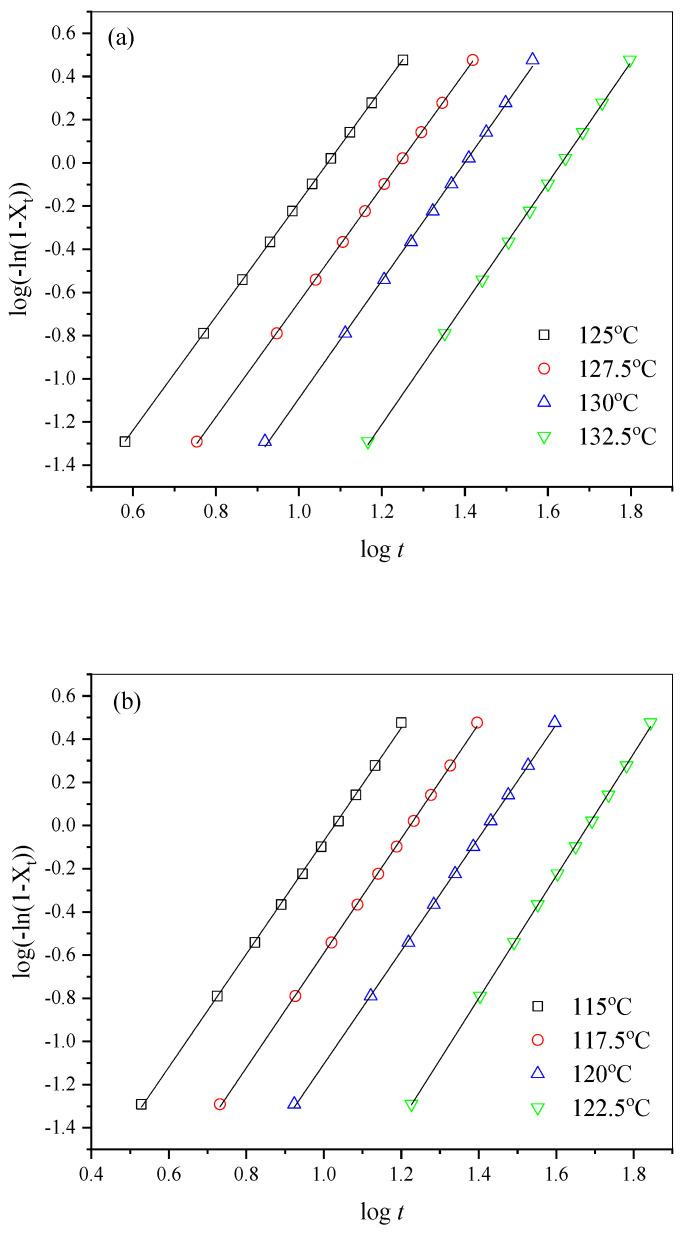

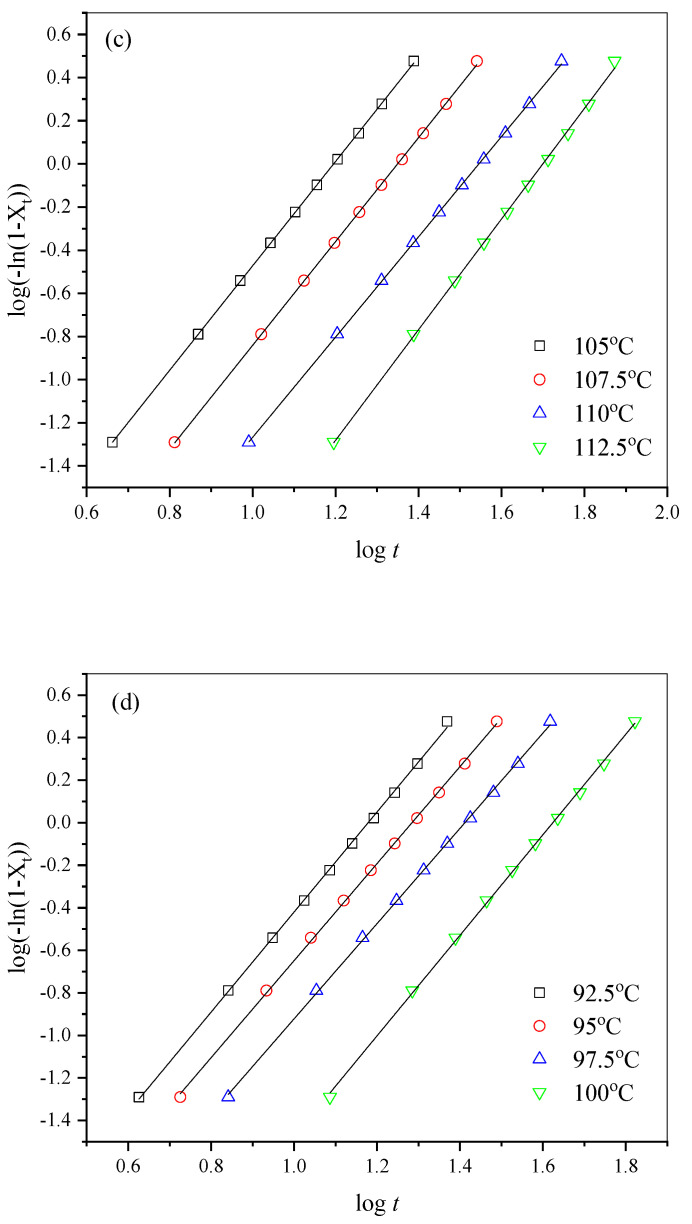
The related Avrami plots for (**a**) PHF, (**b**) PHFSe10, (**c**) PHFSe20, and (**d**) PHFSe30.

The isothermal melt crystallization kinetics were studied by the Avrami equation. The relationship between relative crystallinity (*X*_t_) and crystallization time (*t*) may be described as follows:

(1) $$1\;-{\;X}_{t}=\exp\;(-{kt}^{n})$$

where *n* is the Avrami exponent, and *k* is the crystallization rate constant related to both nucleation process and crystal growth [40,41]. The Avrami plots are displayed in Figure 6. The almost-parallel straight lines indicated that the Avrami method fitted the isothermal melt crystallization process of all the samples very well.

From Figure 6, the values of *n* and *k* were acquired and are listed in Table 3. The *n* values slightly varied between 2.2 and 2.8, suggesting that the crystallization mechanism remained unchanged. The *k* values gradually decreased with the increase in *T*_c_, suggesting a slower crystallization rate. For a better comparison, crystallization half-time (*t*_0.5_) was calculated through the following equation:

(2) $$t_{0.5}=(\frac{\ln2}{k}{)}^{\frac{1}{n}}$$

The calculated *t*_0.5_ data are also shown in Table 3. For each sample, *t*_0.5_ gradually increased with the increase in *T*_c_, indicating that the increase in *T*_c_ decreased the crystallization rate.

The subsequent melting behavior was also studied with DSC for PHF and PHFSe copolyesters after they finished the isothermal melt crystallization at the indicated *T*_c_s. Figure S1 in the Supporting Information demonstrates the relevant melting behavior. Both PHF and PHFSe copolyesters displayed a single melting endotherm at each *T*_c_, which gradually increased with *T*_c_.

**Figure 7:**
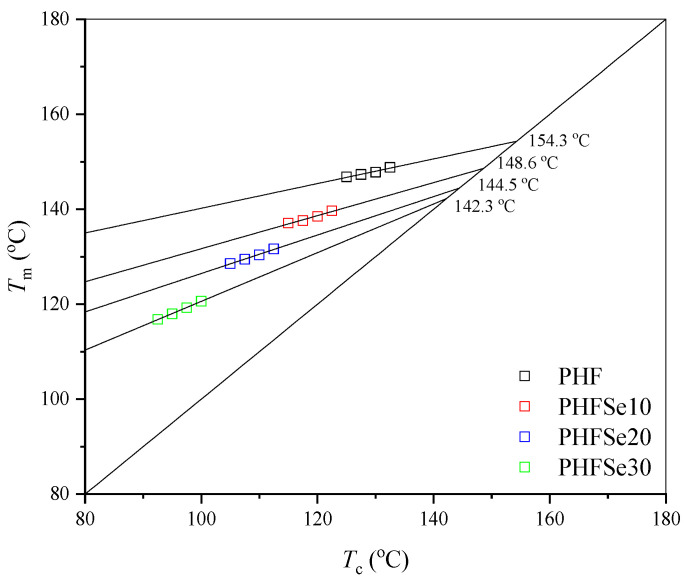
Hoffman−Weeks plots of PHF and PHFSe copolyesters.

The *T*_m_° values of PHF and PHFSe copolymers were further derived through the classic Hoffman−Weeks equation as follows [42]:

(3) $$T_{m}=\left(1\;-\;\eta \right)T_{m}^{o}{+\eta T}_{c}$$

where *T*_m_ represents the apparent melting temperature at *T*_c_, and *η* assumes values between 0 and 1 and is regarded as a measure of the stability, i.e., the lamellar thickness of the crystals undergoing the melting process. Figure 7 demonstrates the Hoffman−Weeks plots for PHF and PHFSe copolyesters. The *T*_m_° values were thus derived to be 154.3, 148.6, 144.5, and 142.3 °C for PHF, PHFSe10, PHFSe20, and PHFSe30, respectively. In brief, with the increase in HSe unit content, the *T*_m_° values accordingly decreased.

**Figure 8:**
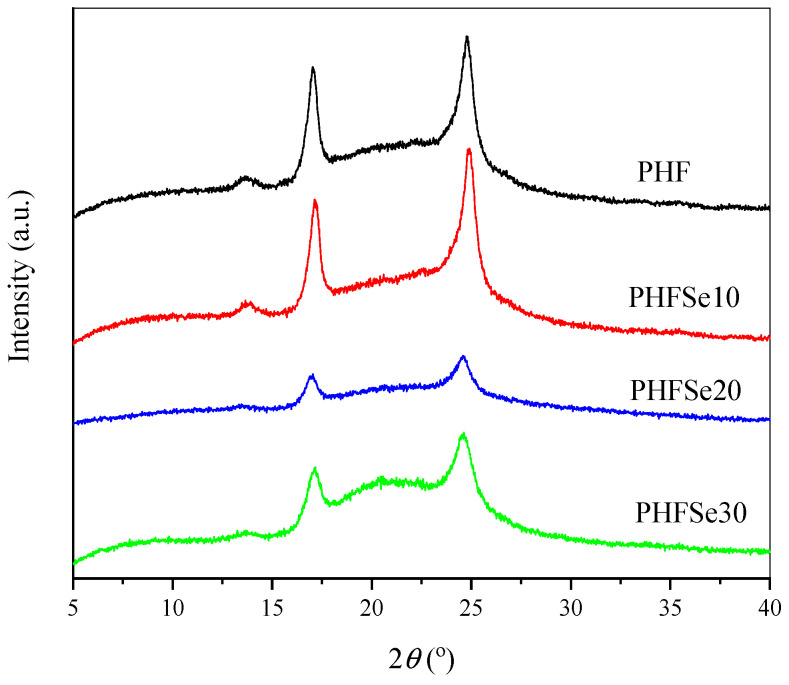
WAXD patterns of PHF and PHFSe copolyesters.

The influence of HSe unit on the crystal structure of PHF was studied by wide-angle X-ray diffraction (WAXD). Figure 8 illustrates the WAXD profiles. PHF showed three characteristic diffraction peaks at 2*θ* = 13.6°, 17.0°, and 24.7°, respectively. In the case of PHFSe copolyesters, they displayed similar WAXD profiles to PHF, suggesting that the presence of HSe unit did not modify the crystal structure of PHF. At present, it is not certain whether the HSe units are totally excluded from the PHF crystals or are partly included into the PHF crystals. Further investigation is necessary to address this issue and will be reported in forthcoming research. In addition, the crystallinity (*X*_c_) of each sample was estimated from Figure 8. The *X*_c_ values of PHF, PHFSe10, PHFSe20, and PHFSe30 were about 43%, 33%, 23%, and 20%, respectively, revealing again that the copolymerization with SeA reduced the crystallinity of PHF.

### 3.3. Tensile Mechanical Property Study

**Figure 9:**
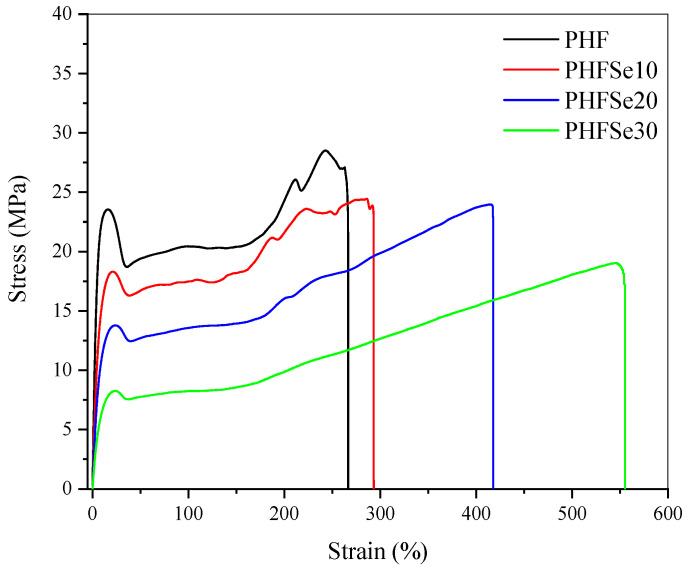
Stress–strain curves of PHF and PHFSe copolyesters.

The influence of low contents of HSe unit on the tensile mechanical property of PHF-based copolyesters was further studied in this section. The stress–strain curves of PHF and PHFSe copolyesters are shown in Figure 9. The increase in HSe unit from 0 to 30 mol% decreased the yield strength (*σ_y_*) from 24.2 ± 0.6 to 8.1± 0.2 MPa, the tensile strength (*σ_b_*) from 30.3 ± 1.4 to 17.3 ± 1.4 MPa, and the Young’s modulus (*E*_t_) from 565.6 ± 23.1 to 114.9±1.5 MPa, respectively. On the contrary, the *ε_b_* increased from 261.6 ± 8.3% for PHF to 513.2 ± 33.8% for PHFSe30, suggesting a remarkably improved toughness. The mechanical property data are summarized in Table 4.

## 4. Conclusions

In this research, three novel, fully biobased PHFSe copolyesters containing low contents of HSe unit from 10 to 30 mol% were successfully synthesized through a common melt polycondensation method from all the biobased monomers, as evidenced by the ^1^H NMR results. Both PHFSe copolyesters and PHF had relatively high and similar intrinsic viscosity values of 0.62 dL/g and above. The thermal behavior (including thermal stability, basic thermal parameters, and crystallization behavior) and mechanical property of PHFSe copolyesters were systematically reported and compared with those of the PHF homopolymer. Regardless of the HSe unit content, both PHFSe copolyesters and PHF showed the high thermal stability. The glass transition temperature, melting temperature, and equilibrium melting temperature gradually decreased by increasing the HSe unit content due to the presence of the flexible HSe segment with a high chain mobility. The copolymerization with SeA reduced the crystallizability of PHF. For instance, the melt crystallization temperature decreased from 97.2 °C for PHF to 51.8 °C for PHFSe30 during a cooling process from the melt at 10 °C/min. The isothermal melt crystallization kinetics study indicated that all samples crystallized more slowly, with an increasing crystallization temperature due to the decrease in the degree of supercooling. The presence of HSe unit did not change the crystal structure of PHF. The tensile mechanical property was further studied, which obviously varied with the HSe unit content. For instance, the elongation at break obviously increased from 261.6 ± 8.3% for PHF to 513.2 ± 33.8% for PHFSe30. In brief, the thermal and mechanical properties of PHF-based copolyesters may be easily tuned by controlling the HSe unit content in this contribution to meet various practical end-use requirements.

## Figures and Tables

**Table 1:** Compositions and [*η*] of PHF and PHFSe copolyesters.

Samples	DMFD/SeA in Feed (mol%)	HF/HSe in Copolyesters (mol%)	[*η*] (dL/g)
PHF	100/0	100/0	0.63
PHFSe10	90/10	90/10	0.68
PHFSe20	80/20	81/19	0.62
PHFSe30	70/30	71/29	0.63

**Table 2:** Basic thermal behavior data of PHF and PHFSe copolyesters.

Samples	*T*_d_ (°C)	*T*_max_ (°C)	*T*_g_ (°C)	*T*_ch_ (°C)	Δ*H*_ch_ (J/g)	*T*_m_ (°C)	Δ*H*_m_ (J/g)	*T*_cc_ (°C)	Δ*H*_cc_ (J/g)	*T*_m_° (°C)
PHF	374.6	393.6	12.3	55.4	12.3	147.4	48.1	97.2	52.6	154.3
PHFSe10	379.4	401.9	−0.9	40.4	4.5	136.8	42.7	89.2	47.2	148.6
PHFSe20	379.8	403.3	−8.4	33.1	23.6	126.0	41.2	69.2	42.0	144.5
PHFSe30	385.1	408.1	−21.0	22.3	22.5	111.2	35.3	51.8	36.5	142.3

**Table 3:** Summary of the Avrami parameters for PHF and PHFSe copolyesters.

Samples	*T*_c_ (°C)	*n*	*k* (min^−*n*^)	*t*_0.5_ (min)
PHF	125.0 127.5 130.0 132.5	2.6 2.7 2.7 2.8	1.50 × 10^−3^ 4.93 × 10^−4^ 1.50 × 10^−4^ 2.72 × 10^−5^	10.21 15.21 21.93 37.68
PHFSe10	115.0 117.5 120.0 122.5	2.6 2.7 2.6 2.8	2.04 × 10^−3^ 5.69 × 10^−4^ 1.88 × 10^−4^ 1.70 × 10^−5^	9.26 14.54 22.95 42.27
PHFSe20	105.0 107.5 110.0 112.5	2.4 2.4 2.3 2.6	1.28 × 10^−3^ 5.66 × 10^−4^ 2.56 × 10^−4^ 4.39 × 10^−5^	13.48 19.21 30.00 43.42
PHFSe30	92.5 95.0 97.5 100.0	2.4 2.3 2.2 2.4	1.69 × 10^−3^ 1.17 × 10^−3^ 6.88 × 10^−4^ 1.45 × 10^−4^	12.91 16.39 21.92 36.02

**Table 4:** Summary of the mechanical property of PHF and PHFDGA copolyesters.

Samples	*E*_t_ (MPa)	*σ_y_* (MPa)	*ε_y_* (%)	*σ_b_* (MPa)	*ε_b_* (%)
PHF	565.6 ± 23.1	24.2 ± 0.6	16.2 ± 1.2	30.3 ± 1.4	261.6 ± 8.3
PHFSe10	320.0 ± 10.3	19.1 ± 0.8	21.9 ± 0.3	24.4 ± 0.4	274.6 ± 18.5
PHFSe20	205.7 ± 8.5	13.7 ± 0.2	23.0 ± 1.4	23.6 ± 2.4	435.8 ± 18.2
PHFSe30	114.9 ± 1.5	8.1 ± 0.2	22.3 ± 1.9	17.3 ± 1.4	513.2 ± 33.8

## Data Availability

The data presented in this study are available on request from the corresponding author.

## Supplementary Materials

The following supporting information can be downloaded at: https://www.mdpi.com/article/10.3390/polym15010085/s1, Figure S1: Melting behavior of (a) PHF, (b) PHFSe10, (c) PHFSe20, and (d) PHFSe30 after isothermal crystallization at indicated *T*_c_s.
