# Supplementary material for: Synthesis, Thermal Behavior, and Mechanical Properties of Fully Biobased Poly(Hexamethylene 2,5-Furandicarboxylate-Co-Sebacate) Copolyesters
Source: Polymers (Basel). 2022 Dec 25;15(1):85. doi: 10.3390/polym15010085 (PMC9823706; doi:10.3390/polym15010085)
Supplement: Supplementary file 1 [file polymers-15-00085-s001.zip › polymers-2051167-supplementary.pdf]

Synthesis, thermal behavior, and mechanical property of fully biobased poly(hexamethylene 2,5-furandicarboxylate-*co*-sebacate) copolyesters

Shiwei Feng, Zhiguo Jiang,* Zhaobin Qiu*

State Key Laboratory of Chemical Resource Engineering, Beijing University of Chemical Technology, Beijing 100029, China

E-mail: jiangzg@mail.buct.edu.cn (Z Jiang)

E-mail: qiuzb@mail.buct.edu.cn (Z Qiu)

Materials

The monomers HDO (purity 99.5%), SeA (purity 98%), and DMFD (purity 99%), were purchased from Shanghai Macklin Biochemical Co., Ltd., Tianjin Fuchen Chemical Reagent Factory, and Ningbo Institute of Materials Technology and Engineering, Chinese Academy of Sciences, respectively. The catalyst TBT (purity 99%) was obtained from Beijing Changping Jingxiang Chemical Reagent Factory. Without further purification, they were directly used as received.

Synthesis of PHFSe copolyesters

The PHFSe copolyesters were prepared from DMFD, SeA, and HDO by the two-step melt polycondensation method, In the esterification/esterification stage, DMFD, SeA, and HDO were put into a three-neck flask at a certain ratio (DMFD+SeA/ HDO=1:1.2), and TBT (0.1 mol% of DMFD) was also added. The mechanical stirring rate was kept at 200-300 r/min under nitrogen atmosphere, and the temperature was increased to 220 ºC for 2 h until no methanol and water were produced. In the condensation stage, the vacuum device was replaced, and the pressure of the device was lowered to 50-100 Pa. The temperature was slowly increased to 235 ºC, and the reaction was carried out at this temperature for 3.5 h until the viscosity of the system was too large for mechanical stirring.

Characterizations

The chemical structure and composition of PHF and PHFSe copolyesters were determined by Nuclear magnetic resonance (^1H NMR) using deuterated chloroform ($CDCl_3$) as the solvent.

The intrinsic viscosity ([η]) values were measured at 25 °C by an Ubbelohde viscometer using chloroform as the solvent.

The thermal stability was studied under nitrogen atmosphere by a TA Q50 thermogravimetric analysis (TGA) at a heating rate of 20 °C/min.

The thermal behavior tests were carried out on a TA Q100 differential scanning calorimeter (DSC) under nitrogen atmosphere. For the T_g and T_m study, the experimental steps were as follow: First, the samples were heated to 190 °C at 40 °C/min to erase the previous thermal history, then cooled to −80 °C at 60 °C/min, and finally heated to 190 °C again at 20 °C/min. For the melt crystallization behavior study, a cooling rate of 10 °C/min was used after the thermal history was completely erased as described above. As for the isothermal melt crystallization kinetics and subsequent melting behavior study, the samples were heated to 190 °C at 10 °C/min after completing the isothermal crystallization at indicated crystallization temperatures.

The crystal structure study was performed on a Rigaku Ultima IV X-ray diffractometer (from 5° to 55 ° at 5 °/min). The samples were isothermally crystallized at indicated temperatures for 10 h.

The tensile properties were measured at room temperature with a tensile rate of 20 mm/min on a universal tensile testing machine (UTM5205XHD) after the samples were hot-pressed and cut into dumbbell shape. The data were obtained from the average values of at least 3 tests.

Melting behavior of PHF and PHFSe copolyesters after isothermal crystallization

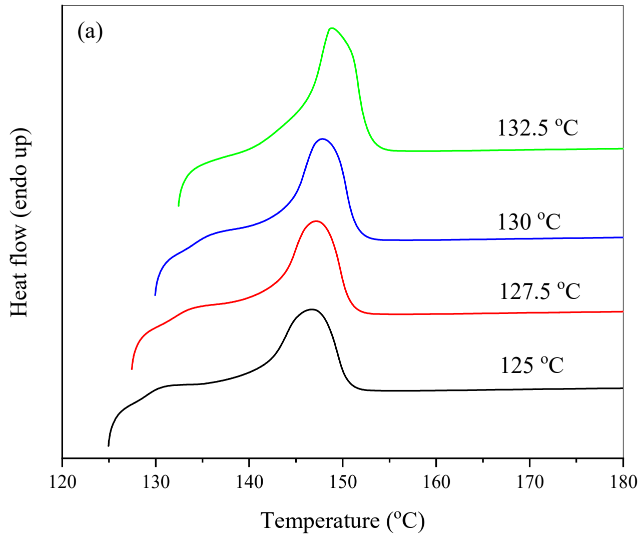

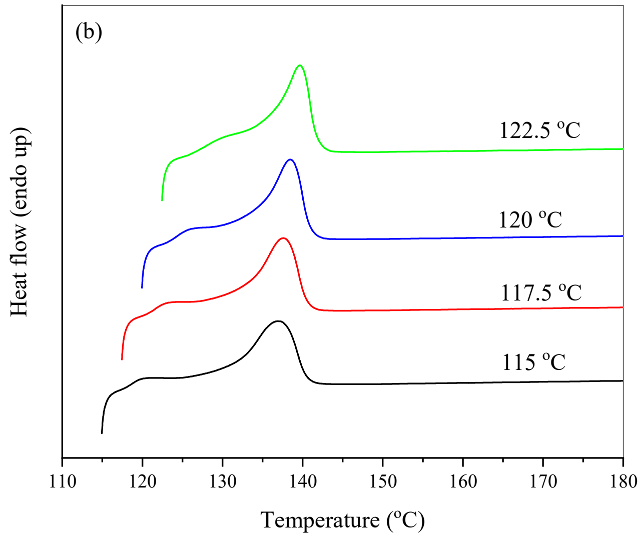

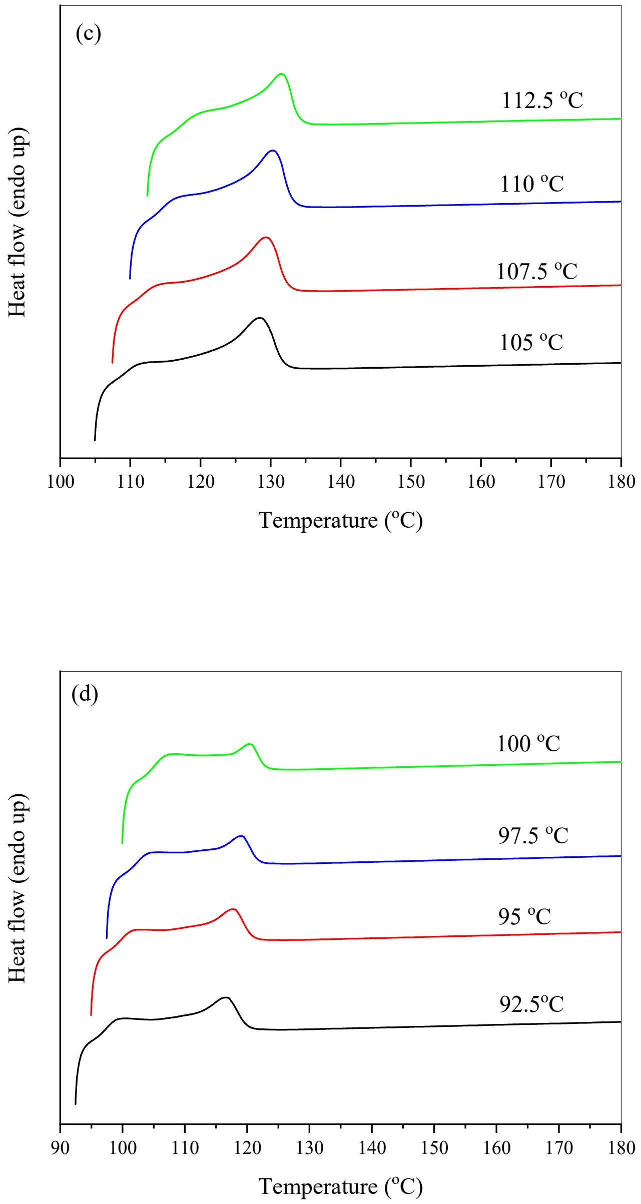

Figure S1. Melting behavior of (a) PHF, (b) PHFSe10, (c) PHFSe20, and (d) PHFSe30 after isothermal crystallization at indicated T_cs.
